# Mobility and International Collaboration: Case of the Mexican Scientific Diaspora

**DOI:** 10.1371/journal.pone.0126720

**Published:** 2015-06-05

**Authors:** Rafael Marmolejo-Leyva, Miguel Angel Perez-Angon, Jane M. Russell

**Affiliations:** 1 PhD Program on Science, Technology and Society, Cinvestav-IPN, 07360, Mexico City, Mexico; 2 Physics Department, Cinvestav-IPN, 07360, Mexico City, Mexico; 3 Bibliotecologic and Information Research Institute, UNAM, 04510, Mexico City, Mexico; Université de Montréal, CANADA

## Abstract

We use a data set of Mexican researchers working abroad that are included in the Mexican National System of Researchers (SNI). Our diaspora sample includes 479 researchers, most of them holding postdoctoral positions in mainly seven countries: USA, Great Britain, Germany, France, Spain, Canada and Brazil. Their research output and impact is explored in order to determine their patterns of production, mobility and scientific collaboration as compared with previous studies of the SNI researchers in the periods 1991–2001 and 2003–2009. Our findings confirm that mobility has a strong impact on their international scientific collaboration. We found no substantial influence among the researchers that got their PhD degrees abroad from those trained in Mexican universities. There are significant differences among the areas of knowledge studied: biological sciences, physics and engineering have better production and impact rates than mathematics, geosciences, medicine, agrosciences, chemistry, social sciences and humanities. We found a slight gender difference in research production but Mexican female scientists are underrepresented in our diaspora sample. These findings would have policy implications for the recently established program that will open new academic positions for young Mexican scientists.

## Introduction

The study of scientific Diasporas has received increased interest over the past decade [1, 2, 3]. While the Indian scientific diaspora was first characterized in 1998 by Mahanti et al. [4], the scientific output of Indian researchers working abroad was recently examined by using their production published in mainstream journals [5]. Mobility of Chinese researchers has been found to have a strong impact on their international scientific collaboration [2, 6, 7, 8]. Indian and Chinese scientists working in USA have also contributed to strengthen the technological capacities of their home countries [9]. Such was also the case of Colombian scientists working in Swiss academic and research institutions [10, 11]. The Moldovan scientific diaspora was also studied in this context [12].

The objective of the present paper focuses on the Mexican scientific diaspora through a combination of bibliometric analysis and curricular information on a selected group of 479 Mexican scientists who have been part of the Mexican National System of Researchers (Sistema Nacional de Investigadores, SNI) since 2009 when the SNI opened its program to Mexican scientists working in foreign institutions. However, it is purely an honorific distinction with no economic incentive. While brain drain literature has been concerned mostly with the economic and development impact caused by this migration [13, 14, 15], our interest is focused on the possible impact generated by mobility on the scientific production and collaboration of Mexican researchers. The benefits of combining curriculum vitae (CV) data with bibliometric analysis has been pointed out by Sandström [16] in his study of a group of medical researchers in Sweden. In particular, he found patterns connecting gender, mobility, collaboration and productivity. Even though we did not have access to full CV data of our Mexican diaspora sample, we were able to find interesting patterns among mobility, gender, production and collaboration by combining bibliometric analysis with CVs studies. We were interested also in obtaining productivity and impact differences among several areas of research and country of PhD training in order to compare our results with the data published for local SNI researchers in the periods 1991–2001 [17] and 2003–2009 [18]. These aspects are rarely addressed in the published literature on scientific diasporas. Recently, Basu [19] was able to compare the Indian diaspora output of scientific papers with that of Indian scientists involved in foreign collaborations. However, she used as her diaspora sample a selected set of unique Indian names to search for the diaspora data. In our case, the Mexican diaspora sample was well defined since all researchers kept their Mexican citizenship in order to become members of SNI. It is interesting to note that Basu’s findings are similar to ours since in all cases considered by her: the Indian diaspora published substantially more papers than the Indian scientists involved in foreign collaborations [19].

In the years 1984–1999, Mexican science experienced a period of expansion with new scientific practices and the incorporation of researchers trained abroad, both Mexican and foreign scientists. The National System of Researchers was established in this period [17, 18, 20]. Created in 1984 its purpose was to stop the flow of scientists abroad at the time of severe economic crisis induced by strong devaluations of the local currency. The SNI grants represent on average, 30% of the income of researchers included in the system and is given at different levels depending on the trajectory and research performance of the applicants: candidate level for young researchers and levels I, II and II for more experienced researchers. Given the characteristics of the academic evaluation carried out in the SNI, it is expected that the most productive researchers in Mexico are those represented by the system [18]. However, due to a change in the Mexican governments public policies on science and technology, the opening of new positions in research and academic institutions stagnated in 2000 [21, 22, 23]. As a consequence, a net brain-drain was detected in Mexico by Licea de Arenas et al. [24]. This group of researchers suggested that the Mexican brain-drain might be associated with the lack of ability of the Mexican institutions to absorb and adequately employ PhD students trained abroad. Our results lend weight to this assumption but we also found high mobility associated with scientific productivity and impact greater than that observed for researchers working in local institutions [18, 23]. Mexican diaspora scientists may thus be involved in a more complex globalized economy that has created new opportunities for migrant researchers.

## Methods

The primary data source consisted of 468 Mexican researchers working abroad but recognized as members of the Mexican National Research System (SNI) since 2009. We added another eleven scientists not integrated into the SNI but who had submitted their personal data after a preliminary set of interviews with a view to including them in the present study which are classified as W/O in our Table 1. This makes a set of 479 Mexican scientists working abroad and active in research. Also Table 1 shows the main characteristics of our sample by field of research, membership level in the SNI system, gender and average age. We have grouped this set of researchers according to the ten knowledge fields used in the global indicators of the Atlas de la Ciencia Mexicana: biological sciences, chemical sciences, physics, mathematics, geosciences, engineering, medicine, agrosciences, social sciences and humanities, according to the data publicly available in two comprehensive studies on the Mexican scientific community [18, 23].

**Table 1:** Distribution of researchers in the Mexican Diaspora by knowledge area, gender (numbers in parenthesis correspond to female scientists) age average and academic category in the SNI: w/o level, candidate (C), levels I, II and III.

Area	Researchers	Graduated in Mexico	Graduated Abroad	Average Age	W/O	C	I	II	III
**Biological Sciences**	136 (58)	110 (48)	26 (10)	40		43	83	6	4
**Physics**	67 (15)	43 (12)	24 (3)	39	9	19	36	2	1
**Chemical sciences**	48 (12)	36 (9)	12 (3)	41		18	25	3	2
**Geosciences**	12 (2)	3 (2)	9 (0)	51		3	4	3	2
**Mathematics**	17 (4)	4 (1)	13 (3)	43		2	9	4	2
**Engineering**	63 (10)	21 (4)	42 (6)	42	2	21	36	4	
**Agrosciences**	29 (9)	10 (5)	19 (4)	49		11	15	2	1
**Medicine**	46 (24)	36 (18)	10 (6)	45		4	31	9	2
**Social sciences**	42 (7)	12 (2)	30 (5)	48		15	25	1	1
**Humanities**	19 (10)	5 (4)	14 (6)	53		3	12	2	2
**Total**	479 (151)	280 (105)	199 (46)	45	11	139	276	36	17

Our measures of productivity and impact correspond to the publications and citations in mainstream journals included in the Web of Science (WoS) in the period 2000–2013. The publications were obtained by matching the names of the 479 researchers with the articles from the WoS database. Our search gave 7047 papers published by our diaspora sample in the period 2000–2013. The respective citations were obtained from WoS journals until 2013. It is important to notice that we will compare our bibliometric data with those obtained in the periods 1991–2001 [17] and 2003–2009 [18], which used the WoS and SCOPUS databases, respectively. However, we expect that the use of different mainstream, multidisciplinary databases will not produce significant differences over the time in the production averages of researchers in the fields of biological and exact sciences. We constructed a SPSS data base with the bibliometric information for each one of the authors in the ten research fields included in our diaspora sample. We used the descriptive statistics crosstab method from SPSS, which are in fact contingency tables that allowed us to perform comparisons of relationship/independence among two or more categorical variables, either nominal or ordinal. In these tables the categories of one variable are defined by frequency or category rates (percentages) of a second variable. In order to determine the frequency of each categorical variable, it was necessary to select each variable from the sub-menu of the SPSS descriptive statistics and cross the data directly with the cross tab analysis. The respective results generate collaboration and mobility tables as well as other relevant information on our Mexican diaspora sample.

## The Sample

There is no reliable data on the total number of Mexican researchers working abroad. In 2013 the Mexican Council on Science and Technology (Consejo Nacional de Ciencia y Tecnología, Conacyt) opened a new program designed to hire 600 young scientists with five years grants, similar to the Ramon y Cajal program implemented by Spain 15 years ago [25]. About 3500 researchers applied for these positions but preliminary information indicates that only 12% of these candidates held positions abroad [26]. In this framework, our set of 479 researchers seems to be a representative sample of the Mexican diaspora. We can appreciate in Table 2 that the hard sciences are well represented in our sample (biological sciences, physics, chemical sciences, engineering and medicine) but since social sciences and humanities are the disciplines in the SNI with the largest number of members, these are underrepresented. These two disciplines have also the largest proportion of researchers trained in local universities (55% and 65%, respectively) and it is possible that this circumstance induces a low mobility of scientists out of the country [23].

**Table 2:** Distribution of researchers in the scientific Mexican Diaspora by country where they obtained their PhD degree (2013).

Country where doctoral degree was obtained.	Area of knowledge									
	Biological sciences	Physics	Chemical sciences	Geosciences	Mathematics	Engineering	Agrosciences	Medicine	Social sciences	Humanities
**AUS**	1									
**BEL**						1				
**BRA**		1								
**CAN**			1			4	2	1		
**CHL**							1			
**DNK**					1	1				
**FIN**		1								
**FRA**		1	5	1		3	3	2	3	1
**DEU**		1			1	3	1	1		
**IND**		2				1				
**ITA**										
**JPN**										1
**MEX**	64	43	35	3	4	20	9	35	12	5
**NLD**							1		1	
**RUS**		3				2				
**ESP**	1	1	1	1		5		1	7	3
**ZAF**										1
**SWE**									1	
**CHE**					1					
**GBR**	3	6	3			10	2	1	7	
**USA**	8	8	2	7	9	10	6	4	11	8
**NonSpecified**	59		1		1	3	4	1		
**Total**	136	67	48	12	17	63	29	46	42	19

Most of the researchers in our sample have the lowest membership levels in the SNI (87%, Candidate and Level I), shown in Table 1. The average age of our sample is about 40 years, which is well below the age average of the members of SNI (above 50 years, [18, 23]). These facts may reflect that most of the researchers in our sample are holding postdoctoral positions in foreign institutions. This scenario is also consistent with the likelihood that their memberships in the SNI is linked to their interest in returning home and taking advantage of the SNI economic incentive as soon as they get a position in a Mexican institution. It should be noted that each membership level in the SNI system, “C” or candidate level, level I, level II & level III, implies a monthly income of 3, 6, 8 and 14 minimum wages, respectively, for each researcher in addition to their existing institutional salary.

Table 2 shows the distribution of researchers by country where they obtained their PhD degree and Table 3 their current geographical location. About 50% of the sample obtained their degree in Mexico with the USA as clearly the leading foreign country for training and research residence. The former is consistent with the general trend for training most new Mexican researchers in local institutions according to a comprehensive study on the Mexican scientific community [23]. It is also relevant to note that about one fifth of the sample (103 researchers) returned to Mexico during our study period (Table 3).

**Table 3:** Distribution of the number of researchers in the scientific Mexican Diaspora by present country of residence vs. area of knowledge (2013).

Country of residence	Area of knowledge									
	Biological sciences	Physics	Chemical sciences	Geosciences	Mathematics	Engineering	Agrosciences	Medicine	Social sciences	Humanities
**AUS**	1		2		1					
**AUT**		1	1							
**BEL**	1						1			
**BRA**		1				1				
**CAN**	2		3			1	1	6	1	
**CZE**			2							
**CHL**		1								
**KOR**						1				
**DNK**										
**FIN**										
**FRA**	2	2	4			3	1	1		
**DEU**		4	1		2	6				
**ISR**						1				
**ITA**	1									
**MEX**	44	18	7	1	5	11	5	11		1
**NLD**							1		1	
**PER**									1	
**PRT**						1				
**RUS**										
**ESP**	1	5	2			6			1	1
**SWE**									2	
**CHE**		2	1							
**GBR**	6	4	1		1	2	1	1		
**USA**	42	17	10	5	4	7	6	10		3
**Non Specified**	36	12	14	6	4	23	13	17	36	14
**Total**	136	67	48	12	17	63	29	46	42	19

## Mobility and International Collaboration

**Fig 1:**
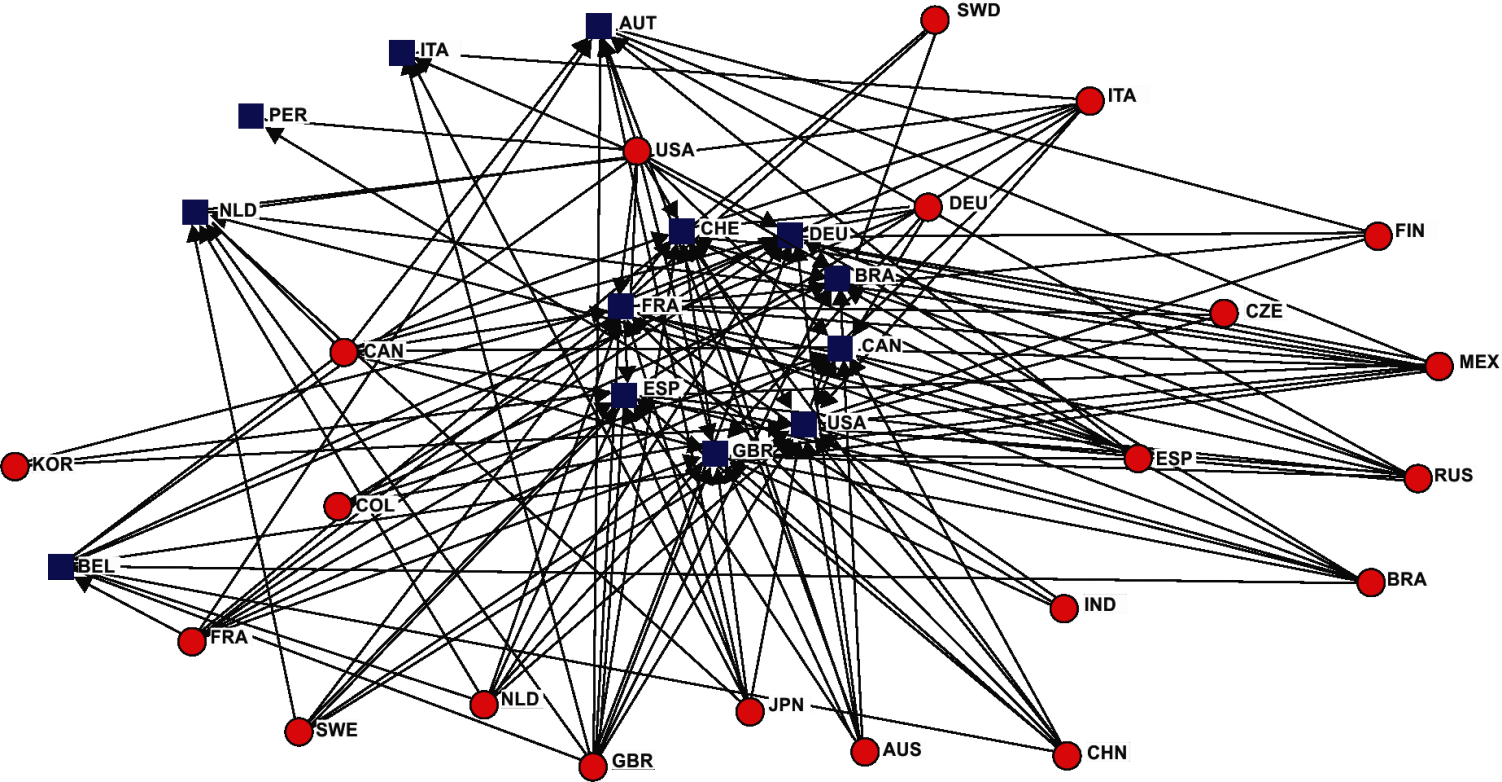
Collaboration Network, using Netdraw from UCINET. We adjust the scaling/ordination from which we select the nearest Euclidian method. **Note:** We have positioned at the center of the "Fig. 1" the seven countries with the most relationships. Red circles correspond to countries of residence and blue squares to countries of scientific collaboration.

The bibliometric analysis of co-authored papers published by the 479 researchers included in our sample has been used to construct a knowledge network among Mexican scientists and a variety of researchers, working in foreign and Mexican institutions. There are a small number of countries that concentrate most of the Mexican diaspora: USA, Great Britain, Germany, France, Canada, Spain and Brazil "Fig 1". In two recent bibliometric studies on international scientific migration [19, 27], it was found that USA, GBR and China were the three main scientific destination for the authors associated with 17 countries, including Mexico. Even though these studies have identified only authors but not national researchers of these countries, it is interesting that our Mexican diaspora sample also has USA and GBR as the main scientific destination.

In Table 4 we present the distribution of co-authored papers published in collaboration by country of residence vs. the country of the main collaboration. We have included also the large number of papers registered in collaboration with Mexican institutions, which show that some researchers of the Mexican diaspora maintain a close relationship with their home country. In Tables 5 and 6 we include the number of co-authored papers published in collaboration for biological sciences and physics, the two most productive areas of research developed by the diaspora scientists. We have restricted the data to just the seven countries in the case of biological sciences and nine in the physical sciences that have the largest concentration of diaspora researchers.

**Table 4:** Distribution of co-authored papers published by the Mexican Diaspora by current country of residence and country involved in the collaboration.

Country of co-authorship	Present country of residence												
	AUT	BEL	BRA	CAN	CHE	DEU	FRA	GBR	ITA	NLD	PER	ESP	USA
**AUS**	1		48	3	62	24		3				6	212
**BRA**		12	175		1	8	10	9				11	785
**CAN**	11	1	8	580	15	2	1	24		2		18	914
**CHN**		2		10	1		4	9				5	335
**COL**			2	10		1							187
**CZE**						5	7						518
**DEU**	2	5		14	18	204	37	9				28	1300
**GBR**	1	7	1	1	10	24	27	450	1	7		65	1522
**ESP**	4			10	4	6	31	17		3		345	645
**FIN**	36					3	48	1					214
**FRA**	1	12		7	6	25	227	9				7	1955
**IND**					0		57	1			1		522
**ITA**		5			8	5	64	24	77	7		32	1214
**JPN**	5				6		36	1	2	2		31	917
**KOR**							5					35	726
**MEX**	98	37	41	313	35	111	177	201		48		265	2093
**NLD**		6				9	5	1		14		2	488
**RUS**	1		1		6	1	26	1				20	1210
**SWD**					1		3						357
**SWE**					8	1	5	4		4		13	229
**USA**	86	9	69	76	126	39	310	77	17	10	79	270	17059

**Table 5:** Distribution of co-authorships for the 2104 papers published by the scientific Mexican Diaspora in the biological sciences by country of residence vs. countries of co-authorship (2000–2013).

Country of co-authorship	Country of residence						
	BEL	CAN	FRA	ITA	ESP	GBR	USA
**BEL**	17					2	
**CAN**		10					52
**ESP**					10	8	13
**FRA**	5		7			8	26
**GBR**				1		105	23
**ITA**				26		8	7
**MEX**	2	2	1			2	168
**USA**	3	3	2	7	3	23	612

**Table 6:** Distribution of co-authorships for the 2007 papers published by the scientific Mexican Diaspora in the physical sciences by country of residence vs. countries of co-authorship (2000–2013).

Country of co-authorship	Country of residence								
	AUT	BRA	CHE	CHL	DEU	ESP	FRA	GBR	USA
**ARG**			2						90
**AUS**		39	52		1	6			0
**BRA**		13	1				6		383
**CAN**		6	12			11			169
**CHN**			1			3	4		143
**COL**									89
**CZE**					3		7		273
**DEU**	2		13	1	33	5	23	1	558
**ECU**									89
**ESP**	1		4	4		33	9		115
**FRA**			5	8		4	34		899
**GBR**		1	10	2	11	15	3	40	407
**IND**				3			21		266
**ITA**			7	5			64		37
**KOR**						2	5		122
**MEX**	11		4	2	3	13	16	6	158
**NLD**							5		237
**RUS**	1		5		1		26		454
**SWD**			1				3		177
**USA**		48	105	9	2	20	36	2	3673

The pattern that emerges from the data shown in Tables 4, 5 and 6 is the close similarity to the trend observed for Mexican collaborations in recent years: a large percentage of papers coauthored with USA and European institutions, and few collaborations with Latin-American scientists [28, 29]. Basu also found that the Indian diaspora has a predominant collaboration with USA and Europe [19].

Tables 7 and 8 include the distribution of papers published by the diaspora researchers in biology and physics and by SNI level. We can appreciate that researchers in level I and Candidate have most of the scientific production in this period. Table 9 shows that researchers in biological sciences graduated in Mexico have more papers and citations than those graduated abroad; the male production is higher in this case. On the contrary, Table 10 shows that physicists graduated abroad produced more papers and citations than the rest of the researchers. The data are presented by gender and country of training. Finally, in Table 11 we have included the number of papers published in the period 2000–2013 by the diaspora researchers in physics and biology with emphasis in the contributions by gender and country of training, while in Table 12 we present the respective averages per year and per researcher. In order to get a measure of the gender difference in productivity, and also by country of training, we have separated the data in Table 11 accordingly. We also include in Table 12 the results obtained in refs. [17] and [18] for the periods 1991–2001 and 2003–2009, respectively, for the SNI members working in local institutions. However, the data in these two references was not worked out fully by gender and country of training.

**Table 7:** Distribution of the average of papers produced by year by the Mexican Diaspora vs. level in the SNI of the biological sciences area.

Level in the SNI system	Biological Sciences															
	*2000*	*2001*	*2002*	*2003*	*2004*	*2005*	*2006*	*2007*	*2008*	*2009*	*2010*	*2011*	*2012*	*2013*	*Mean*	*SD*
**Level 1**	7	12	28	33	51	58	64	116	112	124	134	155	180	88	83.0	54.9
**Level 2**	8	3	4	11	12	13	6	13	17	15	31	22	24	9	13.4	7.9
**Level 3**	19	13	23	21	21	13	15	22	16	18	16	14	15	7	16.6	4.4
**Level C**	3	6	2	8	9	13	25	32	23	32	30	36	35	24	19.9	12.5
**Total**	37	34	57	73	93	97	110	183	168	189	211	227	254	128	33.2	40.2

**Table 8:** Distribution of the average of papers produced by year by the Mexican Diaspora vs. level in the SNI of the physics area.

Level in the SNI system	Physics Area															
	*2000*	*2001*	*2002*	*2003*	*2004*	*2005*	*2006*	*2007*	*2008*	*2009*	*2010*	*2011*	*2012*	*2013*	*Mean*	*SD*
**Level 1**	6	7	9	28	36	71	83	57	41	56	80	72	77	58	48.6	27.7
**Level 2**	6	4	9	1	1	10	8	2	3	1	3	1	2	1	3.7	3.2
**Level 3**	0	0	0	0	0	1	0	0	0	0	0	0	0	0	0.1	0.3
**Level C**	4	2	12	3	5	19	24	39	28	34	21	34	22	22	19.2	12.4
**Total**	31	31	43	46	59	127	134	118	99	150	156	182	164	143	17.9	24.4

**Table 9:** Cites vs. number of papers published by the Mexican diaspora in biological sciences by gender and country of PhD.

Cites	Biological sciences			
	Number of papers of graduates in Mexico		Number of papers of graduates abroad	
	*Feminine*	*Masculine*	*Feminine*	*Masculine*
**0–15**	358	760	112	231
**16–30**	49	124	17	57
**31–45**	25	57	16	21
**46–60**	13	34	6	7
**61–75**	3	13	3	7
**76–90**	1	7	2	2
**91–105**	4	5	0	2
**106–120**	1	4	1	0
**121–135**	0	5	2	1
**136–150**	1	6	3	0
**151–165**	0	2	0	2
**>165**	1	5	1	4
**Total**	456	1022	163	334

**Table 10:** Cites vs. number of papers published by the Mexican diaspora of physics by gender and country of PhD.

Cites	Physics			
	Number of papers of graduates in Mexico		Number of papers of graduates abroad	
	*Feminine*	*Masculine*	*Feminine*	*Masculine*
**0–15**	140	487	43	804
**16–30**	16	96	8	141
**31–45**	6	25	2	75
**46–60**	1	7	2	42
**61–75**	2	12	1	18
**76–90**	1	7	0	9
**91–105**	1	4	0	12
**106–120**	1	2	0	5
**121–135**	1	2	0	4
**136–150**	0	2	0	3
**151–165**	0	1	0	0
**> 165**	0	0	0	25
**Total**	169	645	56	1138

**Table 11:** Distribution of the number of papers published by the researchers in biological sciences and physics during the period 2000–2013.

Year	Number of papers by gender and per area of knowledge							
	Biology				Physics			
	Graduated Abroad		Graduated in Mexico		Graduated Abroad		Graduated in Mexico	
	*10 Women*	*48 Men*	*16 Women*	*62 Men*	*3 Women*	*12 Men*	*21 Women*	*31 Men*
**2000**	6	13	4	14	2	21	3	5
**2001**	2	7	5	20	1	20	4	6
**2002**	2	11	11	34	2	35	1	5
**2003**	4	18	14	35	0	39	3	8
**2004**	3	21	22	48	1	36	3	19
**2005**	7	13	24	56	9	68	17	37
**2006**	6	19	35	56	5	72	18	43
**2007**	14	25	44	111	1	48	12	58
**2008**	13	35	40	83	5	48	13	37
**2009**	12	33	45	105	4	56	18	74
**2010**	16	41	52	103	3	60	15	82
**2011**	14	40	67	116	0	88	20	89
**2012**	13	47	55	143	1	183	22	69
**2013**	12	20	24	74	0	85	17	98
**Mean**	8.9	24.5	31.6	71.3	2.4	61.4	11.9	45.0
**SD**	5.0	12.6	19.7	39.8	2.6	40.9	7.5	33.5
**Total**	124	343	442	998	34	859	166	630

We have separated the production by gender and country of training indicating the total of women and men in each category.

**Table 12:** Distribution of the average number of papers per author/year in Biology and Physics.

Area of knowledge	Average of papers of researchers graduated abroad			Averages of papers of researchers graduated in Mexico			Averages as references indicate	
	*Female*	*Male*	*Global*	*Female*	*Male*	*Global*	SNI [17]	SNI [18]
**Biological sciences**	0.88	0.51	0.58	1.97	1.15	1.32	0.24	0.74
**Physics**	0.81	5.07	4.25	0.58	1.45	1.09	0.22	0.91

The results obtained for our Mexican diaspora correspond to the period 2000–2013, we include the respective results obtained for 1991–2001 [17] and 2003–2009 [18] for the SNI members working in Mexico.

The pattern that emerges from the data included in Table 11 suggests a difference in the scientific productivity of our diaspora researchers with respect to the SNI members working in local institutions. The difference is larger with respect to the averages obtained for local SNI members in 1991–2001 [17]. It is also clear that biology and physics have distinct publication traditions, which is reflected in the various averages included in Table 12. There is another interesting aspect of the data in Table 12: in general terms, one would expect that female productivity is lower than male productivity in biology and physics. However, in our diaspora sample, female productivity in biology is higher than that of the male researchers. In any case, the gender differences in scientific productivity given in Table 12 are not as high as in the period 1991–2001 for local researchers: 0.27 (female) vs. 0.73 (male) [17].

Another point to stress from the productivity averages given in Table 12 is related to the country of training of our diaspora sample. In the study performed for local SNI members in the period 1991–2001 [17], the average for the number of papers published per year for researchers trained in Mexico was higher, 0.508, than the average obtained for SNI researchers trained abroad, 0.271. However, in our case, the physicists trained abroad have a much higher productivity than those trained in Mexico.

The data on the means and standard deviations of the papers published in the period 2000–2013 by researchers in the ten areas of knowledge are shown in Table 13 for all levels in the SNI system. As expected, social sciences and humanities have the lowest scientific productivity measured in terms of the number of papers published in mainstream journals. As was the case with the data shown in Tables 7 and 8 for the biological and physical sciences, the average numbers of papers published per year by the researchers in our diaspora sample is higher than that of the local members of SNI in the years 1991–2001 [17].

**Table 13:** Distribution of means (μ) and standard deviations (Ơ) of papers published by each level of researchers in the SNI system by area of knowledge.

SNI Level	Biological sciences		Physics		Agrosciences		Chemical sciences		Engineering		Geosciences		Mathematics		Medicine		Social sciences		Humanities	
	μ	Ơ	μ	Ơ	μ	Ơ	μ	Ơ	μ	Ơ	μ	Ơ	μ	Ơ	μ	Ơ	μ	Ơ	μ	Ơ
1	83	54.9	48.64	27.73	9.87	7.15	29.85	20.17	40	24.52	1.07	1.1	3.43	3.44	33.33	25.61	5.2	3.97	6.82	5.15
2	13.43	7.94	3.71	3.22	1.73	2.02	9.77	4.68	11.33	6.83	3.8	3.73	6.36	2.95	21.47	11.76	0	0	0.73	0.9
3	16.64	4.36	0.07	0.27	0.33	0.9	3.23	1.83	0	0	8.33	4.03	2.07	2.02	8.47	5.29	0.4	0.7	1.45	1.44
C	19.86	12.53	19.21	12.38	5.13	3.74	7.15	4.47	7.07	7.01	0.93	1.28	1.57	1.4	2.33	3.11	1.9	1.37	0.09	0.3
Total	33.23	40.18	17.91	24.36	4.27	5.51	12.5	14.63	14.6	20.02	3.53	4.12	3.36	3.13	16.4	18.53	1.88	2.91	2.27	3.76

Tables 14 and 15 show the distribution of the number of years spent in various countries by the Mexican diaspora scientists working in biological sciences and physics. The average number of years spent in foreign countries for the biological sciences and physics are 4.3 and 5.7, respectively. Similar averages are observed for the other eight fields of research. These results are consistent with our assumption that most of the Mexican researchers included in our diaspora sample hold or held postdoctoral positions abroad.

**Table 14:** Distributions of researchers in biological sciences by years spent in foreign institutions.

Yearsspent	Countries where researchers were residing/transit										
	BRA	CAN	CHE	DEU	ESP	FRA	GBR	ITA	NLD	SWE	USA
**1**	7	15	13	18	21	18	31	9	5	8	18
**2**	2	2		5	8	7	13	4	5	1	17
**3**	3	4	4	2	6	2	4		2	1	20
**4**	1	3		2	2	2	1	2	1		13
**5**		3			2		3				1
**6**						1	1	1			7
**7**		1	1				2	1			8
**8**						1	3				4
**9**				1							1
**10**			1	1							2
**11**											2
**12**											1
**13**											
**14**											2

**Table 15:** Distributions of researchers in physics by years spent in foreign institutions.

Yearsspent	Countries where researchers were residing/transit														
	AUS	BRA	CAN	CHL	CHN	DEU	ESP	FRA	GBR	ITA	JPN	MEX	RUS	SWE	USA
**2**	6	6	7	7	7	13	13	14	11	8	7	5	6	4	1
**3**	1	1	3	1	1	7	5	4	4	3		3	1	2	4
**4**	1	2	3	1	3	7	5	5	2	3	4	7	1	2	8
**5**	1	2	3		2	7	2	2	6	2	1	1	2	2	5
**6**		1	1		1	2	4	3	2	1		5	1		2
**7**			2	1		1	3	1	3	1	2	15	1	1	3
**8**		1							1			6	1		5
**9**						1					1	2	1		1
**10**						1			1						1
**11**	1								1						1
**12**												1			
**13**															2
**14**												1			
**15**												2	1		
**17**		1													1
**21**		1													
**23**													1		
**29**															1
**30**															1
**33**															1

Tables 16 and 17 include the titles of the journals with the largest number of articles published by the Mexican diaspora in the areas of biological sciences and physics respectively. These journals have high impact factors and most of them are located in the first and second quartile of each category, only a few journals are located in the last quartiles, indicating that the Scientific Diaspora has made important contributions to major scientific journals. To perceive this also we registered the number of citations of all papers produced by each field, and we can appreciate the large numbers of citations generated by them.

**Table 16:** Mainstream journals with the highest number of papers by the Mexican Diaspora in biological sciences with their citations (up to December 2013) and JCR impact factors (2013).

Journals of Biological Sciences Area	Number of Articles	Number of Citations	Impact Factor of the Journal	ISI WoK Category	Maximum Impact Factor per Category	Quartile of the Journal per Category
**BIOPHYSICAL JOURNAL**	87	328	3.83	BIOPHYSICS	12.25	18/74 = 1st
**FASEB JOURNAL**	57	15	5.48	BIOCHEMISTRY& MOLECULAR BIOLOGY	33.116	47/291 = 1st
**PROCEEDINGS OF THE NATIONAL ACADEMY OF SCIENCES OF THE UNITED STATES OF AMERICA**	43	1611	9.8	MULTIDISCIPLINARY SCIENCES	42.351	4/55 = 1st
**JOURNAL OF NEUROSCIENCE**	32	1319	6.75	NEUROSCIENCES	31.376	24/252 = 1st
**PLOS ONE**	31	198	3.53	MULTIDISCIPLINARY SCIENCES	42.351	8/55 = 1st
**JOURNAL OF BIOLOGICAL CHEMISTRY**	29	636	4.6	BIOCHEMISTRY & MOLECULAR BIOLOGY	33.116	65/291 = 1st
**CIRCULATION**	24	148	14.94	CARDIAC & CARDIOVASCULAR SYSTEMS	15.343	2/125 = 1st
**JOURNAL OF BACTERIOLOGY**	21	324	2.68	MICROBIOLOGY	23.317	51/119 = 2nd
**JOURNAL OF PHYSIOLOGY-LONDON**	19	710	4.54	PHYSIOLOGY	29.041	8/81 = 1st
**JOURNAL OF ECONOMIC ENTOMOLOGY**	17	246	1.6	ENTOMOLOGY	13.021	22/90 = 1st
**JOURNAL OF NEUROCHEMISTRY**	16	61	4.24	NEUROSCIENCES	31.376	63/252 = 1st
**CIRCULATION RESEARCH**	15	637	11.089	CARDIAC & CARDIOVASCULAR SYSTEMS	15.343	4/125 = 1st
**JOURNAL OF VIROLOGY**	15	478	4.648	VIROLOGY	12.194	7/33 = 1st
**JOURNAL OF IMMUNOLOGY**	14	381	5.362	IMMUNOLOGY	41.392	24/144 = 1st
**MOLECULAR MICROBIOLOGY**	13	172	5.026	MICROBIOLOGY	23.317	19/119 = 1st

**Table 17:** Mainstream journals with the highest number of papers by the Mexican Diaspora in physics with their citations (closed to December 2013) and the respective JCR impact factors (2013).

Journals of Physics Area	Number of Articles	Number of Citations	Impact Factor of the Journal	ISI WoK Category	Maximum Impact Factor per Category	Quartile of the Journal per Category
**PHYSICAL REVIEW D**	225	5477	4.9	PHYSICS, PARTICLES AND FIELDS	16.53	6/19 = 2nd
**PHYSICAL REVIEW LETTERS**	199	6811	7.7	PHYSICS, MULTIDISCIPLINARY	42.8	6/78 = 1st
**PHYSICS LETTERS B**	194	5641	6.0	PHYSICS, MULTIDISCIPLINARY	42.8	7/78 = 1st
**EUROPEAN PHYSICAL JOURNAL C**	72	1116	5.4	PHYSICS, PARTICLES AND FIELDS	16.53	5/19 = 2nd
**ASTROPHYSICAL JOURNAL**	55	1092	6.3	ASTRONOMY &ASTROPHYSICS	24.037	7/59 = 1st
**JOURNAL OF HIGH ENERGY PHYSICS**	53	263	6.2	PHYSICS, MULTIDISCIPLINARY	42.8	3/27 = 1st
**PHYSICAL REVIEW A**	44	725	3.0	OPTICS	29.95	12/83 = 1st
**NUCLEAR PHYSICS B**	35	3155	3.9	PHYSICS, PARTICLES AND FIELDS	16.53	8/27 = 2nd
**ASTRONOMY & ASTROPHYSICS**	28	301	4.5	ASTRONOMY &ASTROPHYSICS	24.037	13/59 = 1st
**PHYSICAL REVIEW B**	28	267	3.66	PHYSICS, CONDENSED MATTER	36.425	14/67 = 1st
**PHYSICAL REVIEW E**	27	192	2.33	PHYSICS, FLUIDS & PLASMAS	11.26	9/31 = 2nd
**CHEMISTRY AND TECHNOLOGY OF FUELS AND OILS**	25	4	0.14	ENGINEERING, PETROLEUM	1.137	17/19 = 4th
**REVISTA MEXICANA DE FISICA**	17	33	0.3	PHYSICS, MULTIDISCIPLINARY	42.8	71/78 = 4th
**ACS NANO**	16	328	12.033	CHEMISTRY, MULTIDISCIPLINARY	45.66	9/148 = 1st
**JOURNAL OF CHEMICAL PHYSICS**	15	283	3.12	PHYSICS, ATOMIC, MOLECULAR & CHEMICAL	8.711	8/33 = 1st

Our main results suggest that the Mexican Diaspora tends to work in efficient research groups in their respective area of knowledge or discipline. It should be noted that in Table 16 the journals of biological sciences are predominantly in first quartile of each category with only one found in the second quartile. This is in contrast to Table 17 where it can be seen that two physics journals are in fourth quartile. This is related to the practice of Mexican physicists of publishing in the local journal *Revista Mexicana de Física* and another journal related to applied physics problems on fuels and oils.

In Table 16 we observe that biological sciences area has higher impact factors on average than physics area, 5.9 and 4.6, respectively. However, the biological sciences have fewer researchers graduated abroad than physics as mentioned earlier. It is also interesting to note that the diaspora researchers in biology publish in journals from several subject categories which may be related to local health problems addressed by diaspora biologists. Physics on the other hand, concentrate their output in titles in particles, nuclear and atomic physics.

Researchers from the physics area have a greater number of papers co-authored with Mexico and with other countries but have a smaller impact factor average for their papers. However, if we look at citations from both areas, we observe that physics has more citations on average than the biological sciences suggesting more international collaboration and greater visibility.

## Final Remarks and Conclusions

It has been pointed out that international migration and the mobility of human capital may strengthen the scientific capacity of the home countries [5, 9]. This seems to be the case of the Mexican diaspora studied in the present paper. We have presented quantitative evidence that supports the hypothesis that mobility of Mexican researchers had a strong impact on their production and extent of their scientific collaboration (Tables 11 and 12). Our bibliometric analysis of co-authored papers indexed in the WoS points towards a more robust knowledge network than that observed recently for the local scientific community [28, 29]

The findings of the present study suggest that some Mexican scientists maintain their research connections when they return home (Tables 2 and 3 and "Fig 1"). The journals chosen to publish their papers have high impact factors and the respective number of citations reflects a higher impact than the respective local research production (Tables 16 and 17). As a consequence, their average production is higher than that in general of the younger members of the National System of Researchers (SNI).

Our results suggest that diaspora researchers who earned their PhD degrees in Mexico have similar production and impact to those diaspora scientists who earned their degrees abroad. We found also that there is not a big gender difference in research production (Tables 11 and 12) but those Mexican female scientists seems to be under represented in our diaspora sample. The female percentages in the local areas of knowledge are definitely higher than those shown in our diaspora sample according to a comprehensive report on the Mexican science [18, 23].

We found also that there are significant differences among areas of knowledge (Table 11). The most productive researchers correspond to three areas of knowledge: biological sciences, physics and engineering. The diaspora researchers in these three areas also publish in mainstream journals with the highest impact factors (Tables 16 and 17).

In conclusion, our research strongly suggests that while Mexico may be losing a substantial proportion of its most productive young researchers, this diaspora sample is retaining its ties with Mexican institutions and taking advantage of their research connections in order to consolidate their scientific curricula. These findings should be of interest to the Mexican officials in charge of implementing the new program of Conacyt that will tender new academic positions for young Mexican scientists [26].
